# Cetuximab Can Be an Effective and Low-Toxicity Maintenance Treatment Drug in Patients With Metastatic Colorectal Cancer: A Real-World Study of Zhejiang Cancer Hospital

**DOI:** 10.3389/fphar.2021.632076

**Published:** 2021-05-28

**Authors:** Meiqin Yuan, Zeng Wang, Yazhen Zhao, Tingting Feng, Wangxia Lv, Haijun Zhong

**Affiliations:** ^1^ Department of Colorectal Medicine, The Cancer Hospital of the University of Chinese Academy of Sciences (Zhejiang Cancer Hospital), Institute of Basic Medicine and Cancer (IBMC), Chinese Academy of Sciences, Hangzhou, China; ^2^ Department of Pharmacy, The Cancer Hospital of the University of Chinese Academy of Sciences (Zhejiang Cancer Hospital), Institute of Basic Medicine and Cancer (IBMC), Chinese Academy of Sciences, Hangzho, China

**Keywords:** cetuximab, mCRC, maintenance treatment, survival, safety

## Abstract

After initial treatment, maintenance therapy is now commonly used in mCRC patients, which can help patients live longer, have lower side effects, and higher quality of life. The maintenance treatment may include chemotherapy, targeted therapy, or combined with chemotherapy and targeted therapy. But the evidence of cetuximab maintenance is still scant.

**Methods:** We collected real-world data of wild-type RAS unresectable mCRC patients who were treated with cetuximab-based chemotherapy as the first-line therapy between January 2013 and December 2018 at the Zhejiang Cancer Hospital (Hangzhou, China).

**Results:** A total of 177 patients were ultimately included in the study, and 107 patients had progression information in medical records; all patients had survival data. The median OS was 40.9 ms, ORR was 14.7%, and DCR was 73.5%. The subgroup analysis showed that the mOS was better in maintenance patients than in non-maintenance patients (47.1 vs. 28.6 ms, *p* = 0.001), patients with primary tumor resection had better mOS than who did not (47.1 vs. 35.4 ms, *p* = 0.038). In those 107 patients who had progression information, the median PFS was 9 ms, the median OS was 42.6 ms, ORR was 18.7%, and DCR was 84.1%. The subgroup analysis showed that the mPFS and mOS were 11.6 and 47.1 ms, respectively, in the maintenance group, which were significantly better than 6.1 ms and 28.7 ms in the non-maintenance group (*p* = 0.025 and 0.017, respectively). The mPFS and mOS in patients with efficacy evaluation of CR + PR + SD were 10.3 and 47.1 ms, respectively, which is significantly better than 2.8 and 13.5 ms in the PD patients (*p* = 0.012 and <0.001, respectively). The mOS was best in only lung metastases patients (60.9 ms), then only liver metastases patients (47.1 ms), and then in both liver and lung metastases (42.6 ms); the mOS in patients with other organs metastases was the worst (22.4 ms), *p* = 0.022. The mOS in male individuals is better than that in female individuals, 60.99 vs. 29.1 ms, respectively, *p* = 0.042. The primary tumor site and primary tumor resection also affect the OS, primary tumor resection better than did not (not reach the end vs. 35.7 ms, *p* = 0.048), left side better than right side (47.1 vs. 16.6 ms, *p* < 0.001), which is consistent with the literature report. There was no statistical difference in other subgroups.

**Conclusion:** For patients with all RAS wild-type and initially unresectable mCRC who experienced standard first-line cetuximab-based treatment and maintenance treatment that contained cetuximab can significantly improve the mPFS and mOS, and the observed toxicity was mostly mild too. So, we consider that cetuximab can be an effective and safety maintenance drug in mCRC patients.

## Introduction

Colorectal cancer is a major life-threatening disease worldwide (Bray et al., 2018). Systemic therapy (including chemotherapy and targeting therapy) is the most important and effective therapy for patients with metastatic colorectal cancer (mCRC), and the effect of systemic therapy has been greatly improved in recent years. The most effective chemotherapy drugs for mCRC include irinotecan, oxaliplatin, and fluorouracil (de Gramont et al., 2000; Douillard et al., 2000; Goldberg et al., 2004; Tournigand et al., 2004; Goldberg et al., 2006; Cassidy et al., 2008). The use of chemotherapy regimens based on these drugs combined with molecular targeted drugs including cetuximab and bevacizumab to treat mCRC has already become a clinical consensus at the first-line treatment (Heinemann et al., 2014; Venook et al., 2017). The median survival of mCRC patients treated with the above chemotherapy drugs was about 21 months, while the median survival of mCRC patients treated with the combination of molecular targeted drugs was further extended to nearly or even more than 30 months.

After initial treatment, maintenance therapy is now commonly used in mCRC patients, which can help patients live longer, have lower side effects, and higher quality of life. Maintenance treatment may include chemotherapy, targeted therapy, or combined with chemotherapy and targeted therapy (Tournigand et al., 2006; Chibaudel et al., 2009; Waddell et al., 2011; Yalcin et al., 2013; Aranda et al., 2018). Cetuximab is a recombinant, human/mouse chimeric immunoglobulin G1 mAb that binds exclusively to the extracellular domain of the EGFR and results in several different downstream effects, all of which may contribute to the antitumor activity, such as interfering with apoptosis, cell proliferation, angiogenesis, and the metastatic process. Cetuximab can significantly improve the survival in RAS wild mCRC patients, but the role of maintenance is lack of evidence-based medicine basis. MACRO2 (Aranda et al., 2018), a phase II exploratory trial, suggests that maintenance therapy with single-agent cetuximab following mFOLFOX + cetuximab induction could be a valuable option compared with mFOLFOX + cetuximab treatment continuation. In this article, we collected real-world data of patients who accepted cetuximab alone or combined with chemotherapy as maintenance treatment at Zhejiang Cancer Hospital from 2013 to 2018. It may help us know the role of cetuximab in maintenance therapy of mCRC.

## Patients and Methods

Patients with wild-type RAS WT (KRAS exons 2, 3, and 4 and NRAS exons 2, 3, 4) unresectable mCRC who were treated with cetuximab-based chemotherapy as the first-line therapy between January 2013 and December 2018 at the Zhejiang Cancer Hospital (Hangzhou, China) were retrospectively investigated. Chemotherapy regimens were not restricted. The study was approved by the Ethics Committee and Institutional Review Board of Zhejiang Cancer Hospital and was conducted in compliance with the Helsinki Declaration. The main objective of the study should be progression-free survival (PFS), and the secondary endpoints were overall survival (OS), objective response rate (ORR), and disease control rate (DCR).

The follow-up period was defined as the time from diagnosis to the last observation or death. Progression-free survival was defined as the time between the date of the time from randomization until patient’s tumor progression and death. The log-rank test was used to estimate and compare survival. IBM SPSS Statistics 22 statistical software was used to statistically analyze the data.

## Results

### Patient Characteristics

A total of 177 patients were ultimately included in the study: 117 were male and 60 were female, 145 were ≤65 years old and 32 were ≥65 years old, 158 were left-sided and only 19 were right-sided, 60 had primary tumor resection and 117 had not, 77 had liver metastases only, 27 had lung metastases only, nine had both liver and lung metastases, and 64 had other organs metastases. In these patients, 107 patients had progression information in medical records, and all patients had survival data. Patient characteristics are shown in Table 1.

**TABLE 1:** Baseline characteristics of the metastatic colorectal cancer patients.

Variable		All (*n* = 177)	Patients who had progression information (n = 107)	*p*-value
Sex—No. (%)	Male	117 (66.1)	72 (67.3)	0.897
	Female	60 (33.9)	35 (32.7)	
Age—year	Mean ± SD	52.32 ± 28.63	57.26 ± 29.26	—
Age—no. (%)	≤65 years	145 (81.9)	91 (85)	0.519
	≥65 years	32 (18.1)	16 (15)	
Primary tumor site—no. (%)	Right side	19 (10.7)	17 (15.90)	0.269
	Left side	158 (89.3)	90 (84.1)	
Primary tumor resection—no. (%)	Yes	60 (33.9)	47 (43.9)	0.101
	No	117 (66.1)	60 (56.1)	
Organs with metastases—no. (%)	Liver only	77 (43.5)	50 (46.7)	0.947
	Lung only	27 (15.2)	15 (14.0)	
	Both liver and lung	9 (5.1)	6 (5.6)	
	Other organs	64 (36.2)	36 (33.6)	

### Treatment Exposure

In all of 177 patients, 117 accepted CET + FOLFIRI and 60 accepted CET + mFOLFOX6 as the first-line therapy, 82 patients accepted the maintenance treatment after induction chemotherapy, and 95 patients did not. In those 107 patients who had progression information, 70 accepted CET + FOLFIRI and 37 accepted CET + mFOLFOX6 as the first-line therapy, 64 patients accepted the maintenance treatment, and 43 patients did not. The details are shown in Table 2. The maintenance program could be single cetuximab or cetuximab + chemotherapy (including CPT11 and fluorouracil drugs).

**TABLE 2:** Treatment exposure.

Variable		All (*n* = 177)	Patients who had progression information (*n* = 107)	*p*-value
Chemotherapy regimen—no. (%)	CET + FOLFIRI	117 (66.1)	70 (65.4)	0.907
	CET + mFOLFOX6	60 (33.9)	37 (34.6)	
Best overall response—no. (%)	CR	2 (1.1)	2 (1.9)	0.197
	PR	24 (13.6)	18 (16.8)	
	SD	104 (58.8)	70 (65.4)	
	PD	47 (26.5)	17 (15.9)	
Maintenance or not—no. (%)	Maintenance	82 (46.3)	64 (59.8)	0.037
	Non-maintenance	95 (53.7)	43 (40.2)	

### Efficacy

**FIGURE 1:**
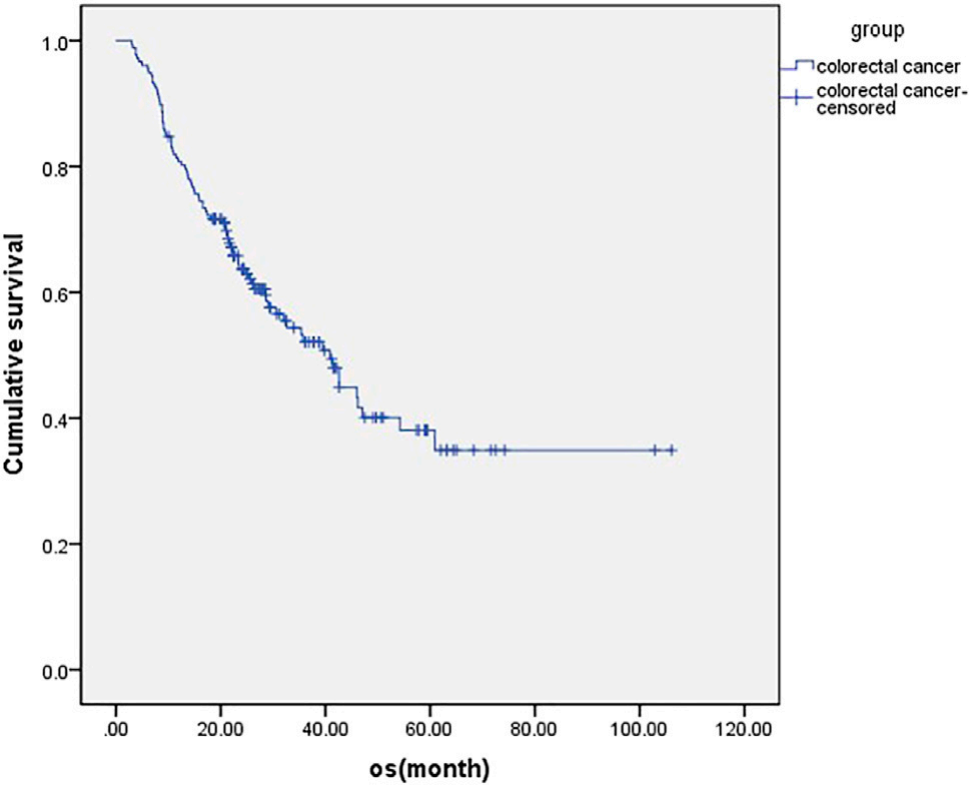
overall survival of all the metastatic colorectal cancer patients (*n* = 177).

**FIGURE 2:**
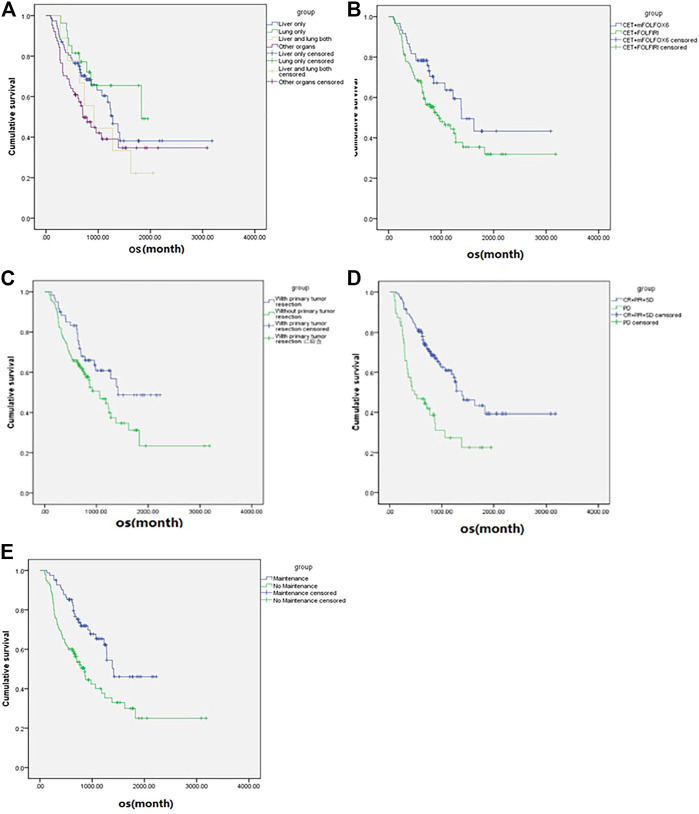
Subgroup analysis (according to the variables in Table 3) of overall survival of the metastatic colorectal cancer patients (*n* = 177). **(A)** Metastases sites. **(B)** Chemotherapy regimen. **(C)** Primary tumor resection. **(D)** Best overall response. **(E)** Maintenance or not.

For all patients included in this study, the median OS was 40.9 ms (Figure 1), ORR was 14.7%, and DCR was 73.5%. The subgroup analysis showed that the mOS was better in the maintenance patients than the non-maintenance group (47.1 vs. 28.6 ms, *p* = 0.001), with primary tumor resection better than did not (47.1 vs. 35.4 ms, *p* = 0.038). There was no statistical difference in other subgroups. The details are shown in Table 3 and Figure 2.

**TABLE 3:** Kaplan–Meier estimates of PFS and OS.

Variable		All (n)		Patients who had progression information(n)			
—		OS	*p*-value	PFS	*p*-value	OS	*p*-value
Sex	Male	41.4	0.753	10.2	0.149	60.99	0.042
	Female	30.6		7.5		29.1	
Age	Mean ± SD	52.32 ± 28.63	—	57.26 ± 29.26	—	—	—
	≤65 years	40.9	0.810	9.2	0.694	42.6	0.703
	≥65 years	34.5		8.5		46.2	
Primary tumor site	Right side	39.4	0.421	3.8	0.106	16.6	0.000
	Left side	46.2		10.3		47.1	
Primary tumor resection	Yes	47.1	0.038	11.8	0.051	—	0.048
	No	35.4		7.7		35.7	
Organs with metastases	Liver only	42.6	0.084	9.4	0.594	47.1	0.022
	Lung only	60.9		11.2		60.9	
	Both liver and lung	30.6		11.0		42.6	
	Other organs	23.5		7.7		22.4	
Chemotherapy regimen	CET + FOLFIRI	32.0	0.054	8.0	0.176	41.4	0.126
	CET + mFOLFOX6	46.2		9.2		54.2	
	PD	15.9		2.8		13.5	
Maintenance or not	Maintenance	47.1	0.001	11.6	0.025	47.1	0.017
	Non-maintenance	28.6		6.1		28.7	

**FIGURE 3:**
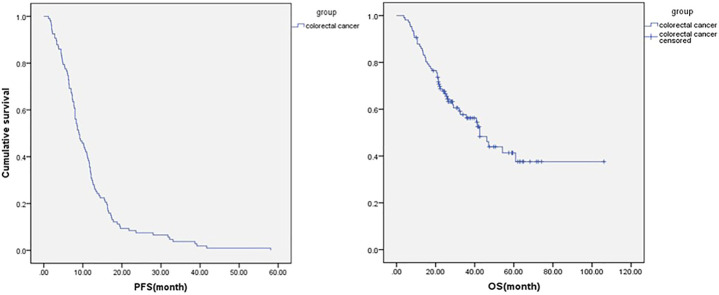
Progression-free survival and overall survival in all metastatic colorectal cancer patients who had progression information (*n* = 107).

**FIGURE 4:**
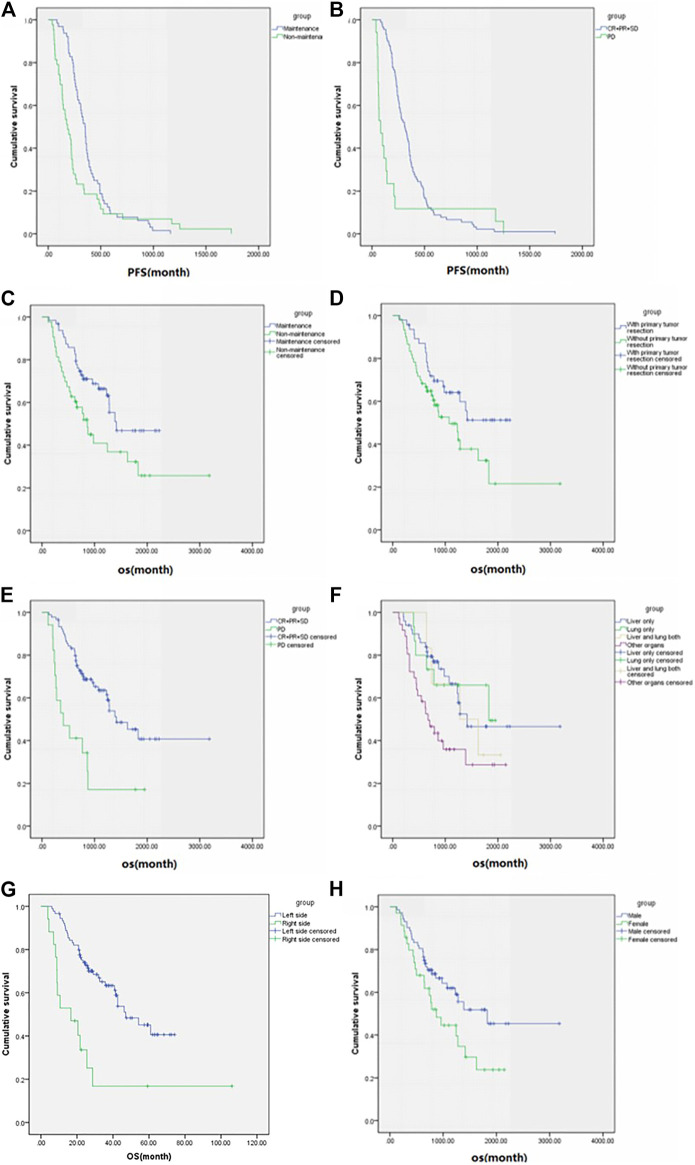
Subgroup analysis (according to the variables in Table 3) of progression-free survival and overall survival in patients who had progression information (*n* = 107). Progression-free survival of **(A)** maintenance or not and **(B)** best overall response and overall survival of **(C**) maintenance or not, **(D)** primary tumor resection, **(E)** best overall response, **(F)** metastases sites, **(G)** primary tumor resection and **(H)** sex.

In those 107 patients who had progression information, the median PFS was 9 ms, the median OS was 42.6 ms (Figure 3), ORR was 18.7%, and DCR was 84.1%. The details are shown in Table 2. The subgroup analysis showed that the mPFS and mOS was 11.6 and 47.1 ms, respectively, in the maintenance group, which were significantly better than 6.1 and 28.7 ms in the non-maintenance group (*p* = 0.025 and 0.017, respectively). The mOS was best in only lung metastases patients (60.9 ms), then only liver metastases patients (47.1 ms), and then in both liver and lung metastases (42.6 ms); patients with other organs metastases was the worst (22.4 ms), *p* = 0.022. The mOS of male patients is better than that in female patients, 60.99 vs. 29.1 ms, respectively, *p* = 0.042. The primary tumor site and primary tumor resection also affect the OS, primary tumor resection better than did not (not reach the end vs. 35.7 ms, *p* = 0.048), left side better than right side (47.1 vs. 16.6 ms, *p* < 0.001), which is consistent with the literature report. There was no statistical difference in other subgroups. The details are shown in Table 3 and Figure 4.

### Safety

In general, the observed toxicity was mostly mild in maintenance patients, and no patients experienced grade 3/4 adverse events including hematology (including leukopenia, anemia, and thrombocytopenia) and non-hematologic toxicity (like skin toxicity, diarrhea, fatigue, and weight loss) in the period of maintenance treatment. Also, no deaths occurred.

## Discussion

Metastatic colorectal cancer (mCRC) is a major healthcare problem worldwide. After initial treatment, maintenance therapy is now commonly used in mCRC patients, which can help patients live longer, have lower side effects, and higher quality of life. The maintenance treatment may include chemotherapy, targeted therapy, or combined with chemotherapy and targeted therapy. Clinical trials have already confirmed that 5-FU, capecitabine, bevacizumab, and bevacizumab plus 5-FU/capecitabine, all can be effective maintenance treatment strategies in mCRC patients. Also, when we mention about targeted maintenance, bevacizumab was always the first and evidence-based option because of the results according to clinical trials, including Stop and Go, MACRO, and CAIRO3. But the role of cetuximab in maintenance is the lack of the evidence-based medicine basis. The phase II exploratory trial MACRO2 suggested that cetuximab, though with little evidence, could be a valuable option as maintenance therapy following mFOLFOX + cetuximab induction (Arm-A) compared with mFOLFOX + cetuximab treatment continuation (Arm-B). PFS at 9 months showed noninferiority between arms (Arm-A/Arm-B: 60%/72%). There were no statistically significant differences in the PFS (Arm-A/Arm-B: 9 months/10 months) or overall survival (23 months/27 months) between arms. The objective response rate was also similar (48/39%). The safety profile was similar between arms too. But the study could not prove the superiority of cetuximab as maintenance therapy than observation only. So, the evidence of cetuximab maintenance is still scant.

In this study, we found that initially unresectable mCRC patients with RAS wild-type and who underwent standard first-line cetuximab-based treatment as well as the maintenance treatment that contained cetuximab can significantly improve their mPFS and mOS, and the observed toxicity was mostly mild too. So, we consider cetuximab can be an effective and safety maintenance drug in mCRC patients. But in our study, all of cetuximab was complimentary by China Charity Federation because of its high cost. Now cetuximab is covered by Medicare and has been reduced in price, but there is no charity policy, so we think that an economic efficacy assessment may be required for the long-term maintenance use.

## Data Availability

The raw data supporting the conclusion of this article will be made available by the authors, without undue reservation.

## Abbreviation

CR, complete response; PR, partial response; SD, stable disease; PD, progressive disease.
